# Supplementary material for: Modelling membrane reshaping by staged polymerization of ESCRT-III filaments
Source: PLoS Comput Biol. 2022 Oct 17;18(10):e1010586. doi: 10.1371/journal.pcbi.1010586 (PMC9612822; doi:10.1371/journal.pcbi.1010586)
Supplement: S1 Appendix — (PDF) [file pcbi.1010586.s001.pdf]

S1 Electron microscopy

Protein purification is performed as described in Pfitzner et al. [1]. For LUV preparation, DOPC:DOPS:Rhodamine-PE (6:4:1; 10 mg/ml) mixture is evaporated in a glass tube, 500 μL of buffer are added, the tube is vortex followed by 5 times freezing and thawing. LUVs are then diluted 1:100 in buffer (20 mM Tris pH.6.8, 200 mM NaCl and 1 mM $MgCl_2$), spun down (10', 5,000 g), resuspended in 4.5 M Snf7 for 6h (4°C), before 1 μM Vps2, 1 μM Vps24, 2 μM Did2 and 2 μM Ist1 are added. Following overnight incubation at 4°C, all samples are spun down 10' 5,000 g and resuspended in buffer (negative stain EM) or buffer containing 30% glycerol (cryofreeze-fracture). Negative stain samples are absorbed onto EM grids and stained with 2% uranyl acetate for 30 s. Freeze fracture samples are transferred onto sample stamps, flash-frozen and processed using a 060 Freeze-Fracture System (BAF). Images are acquired on a Tecnai G2 Sphera (FEI) electron microscope.

Reference for S1 Appendix

[1] Pfitzner AK, Mercier V, Jiang X, Moser von Filseck J, Baum B, Šarić A, et al. An ESCRT-III Polymerization Sequence Drives Membrane Deformation and Fission. Cell. 2020;doi:10.1016/j.cell.2020.07.021.
