# Supplementary material for: Modelling membrane reshaping by staged polymerization of ESCRT-III filaments
Source: PLoS Comput Biol. 2022 Oct 17;18(10):e1010586. doi: 10.1371/journal.pcbi.1010586 (PMC9612822; doi:10.1371/journal.pcbi.1010586)
Supplement: S2 Appendix — (PDF) [file pcbi.1010586.s002.pdf]

S2 Simulation protocols

S2.1 General settings

The three individual ESCRT-III filaments are each modelled using the 3-beaded model described in Harker-Kirschneck et al. [1]. The Flat Spiral has a target geometry of a flat ring which is bound to the membrane at an angle of 90° with respect to its axis of curvature ($\tau = 0°$ following the original notation in [1]). The Wide Helix and the Tight Helix have target geometries of tilted rings (i.e., no pitch is applied in the target geometries) which are bound to the membrane at an angle of 0° with respect to their axes of curvature ($\tau = 90°$ following the original notation in [1]). All neighbouring subunits within a filament are connected to each other via nine harmonic bonds with default bond constant $k = 256\ k_{\mathrm{B}}T/\sigma^2$, where σ is the MD unit of length, unless stated otherwise. This determines the target geometry of the filament.

The initial structure of the membrane is a flat layer of membrane beads arranged in a hexagonal lattice. The membrane is modelled in its fluid state via the one-bead-thick model developed by Yuan et al. [2], with $\epsilon = 4.34\ k_{\mathrm{B}}T, \mu = 3.0, \xi = 4.0, \theta_0 = 0°$, which yields a fluid membrane of bending rigidity $\sim 20k_{\mathrm{B}}T$. Short-ranged 12-6 Lennard-Jones (LJ) interactions are applied between the two bottom beads of the filament's three-beaded subunits and the membrane beads. The membrane binding affinity of all the filaments is kept the same. Short-ranged LJ attractions are also applied at the interfaces of different filaments, through their neighbouring bottom beads (i.e. the inner bottom bead of the Flat Spiral with the outer bottom bead of the Wide Helix and the inner bottom bead of the Wide Helix with the outer bottom bead of the Tight Helix). The attraction is weak enough to allow detachment. The rest interactions within filament beads are volume exclusions.

For simulations with a generic cargo, we place the cargo particle in the centre of the filaments. A weak short-ranged LJ interaction is applied between the cargo particle and the membrane beads, with the interaction strength $\epsilon_{\mathrm{memb-cargo}} = 0.6\ k_{\mathrm{B}}T$. This allows the cargo to stay attached to the membrane, resisting fluctuations, but to not bud spontaneously in the absence of the filament. Volume exclusion is imposed between the cargo particle and the filament beads.

The radii of the coarse-grained beads used in our simulations are $r_{\mathrm{subunit}} = r_{\mathrm{membrane}} = 0.5\ \sigma, r_{\mathrm{cargo}} = 8.0\ \sigma$, where one subunit bead refers to one bead in the 3-beaded protein subunit. A conversion of $\sigma = 2.3$ nm is used to map the MD lengths to physical lengths [1]. All the short-ranged LJ interactions are cut-off at $r = 1.3\ r_{\mathrm{min}}$, where r_{min} is the distance at the potential minimum, and are shifted to zero at the cut-off. The default interaction strength $\epsilon = 3.0\ k_{\mathrm{B}}T$ is used to model all short-ranged LJ interactions for filament-membrane and filament-filament binding, unless specified otherwise. This interaction strength and cut-off range is strong enough to ensure filament-membrane association, but weak enough so that specific filament beads can interact with different membrane beads; thus filaments can slide over the membrane. All the volume-exclusion interactions are treated as LJ interactions truncated at the potential minimum and shifted to zero, with interaction strength $\epsilon = 2.0\ k_{\mathrm{B}}T$. All the interaction parameters are summarized in S1 Table.

The parameters in our model are the following: the radii of the three filaments, rigidities of the three filaments, the filament length, the strength of filament-membrane adhesion, and the diameter of the cargo particle. While we are not modelling a specific system, the geometrical parameters that we choose are in the regime of physiologically-relevant parameters. The target radii of the filaments (30 nm for Flat Spiral, 28 nm for Wide Helix) are based on the AFM measurements reporting Snf7 spirals to be 20-50 nm wide on membrane substrates [3, 4], and the cargo diameter (37 nm) is based on the reported ILV and exosomes diameters of 30-160 nm. The target radius of Tight Helix is chosen by a parameter scan, and it matches well with the theoretical fission limit ($\approx$ 3nm) [5]. The rigidities of the first two filaments are selected such that they yield the observed intermediate conical deformation [6] and, for simplicity, we use the same stiffness for the third filament. Little is known about the strength of the membrane adhesion of these composite filaments. The filament-membrane adhesion is chosen as the smallest value that allows the filaments to remain stably attached to the membrane. This parameter was previously tested when the single filament model was originally developed [1].

The simulations are run with molecular dynamics (MD) in the isothermal-isobaric ensemble with the

barostat targeted at zero pressure. The barostat is applied in coupled x and y directions with damping factor of 10τ, where τ is the MD time unit. Langevin thermostat is applied at each MD step with the temperature set to 1 and the damping coefficient set to 1. Periodic boundary conditions are applied in the x and y directions. A time step of 0.01τ is used. All MD simulations are carried out with LAMMPS code [7] and reduced units are used during the simulation. The unit conversion factors are summarized in S2 Table.

S2.2 Simulation of staged recruitment and disassembly of three filaments with cargo

We first equilibrate a generic cargo by itself on the membrane before adding the ESCRT-III filaments to the system. The membrane is constructed of 29440 beads (160×160 σ^2) and is equilibrated for 10^4 steps, while the cargo and the filaments are kept frozen. Then we allow the cargo to move and equilibrate it for 10^6 steps on the membrane, before placing the three ESCRT-III filaments around it in the shape of a three-stranded flat spiral. Each filament contains 84 subunits.

Although the copolymer is composed of three different filaments, at this stage, only the Flat Spiral is activated. Its target geometry is a flat ring with a target radius of 30.8 nm. The two inner filaments also behave like the Flat Spiral, thereby effectively remaining deactivated. The Wide Helix follows the same target geometry as the Flat Spiral, but with a slightly smaller target radius of 28.0 nm, since it is initially closely packed inside the Flat Spiral. This deactivation is further achieved, by temporarily turning the two inner filaments into one, by coupling their rigid body subunits (i.e., one subunit from the Wide Helix and one from the Tight Helix form a rigid body). This means the Tight Helix now behaves as if it were part of the Wide Helix (i.e., no target geometry for the Tight Helix), rather than an independent filament. We carry out the simulation for 10^6 steps.

To simulate the first copolymerisation, we activate the Wide Helix by changing its target geometry from a flat to a tilted ring, that is 30.8 nm wide, while keeping the Tight Helix as part of it. After another 10^6 steps, the Flat Spiral is disassembled instantaneously. This is modelled by deleting the harmonic bonds between protein subunits and resetting the membrane-filament interactions from short-ranged LJ interactions to volume exclusions.

After another 10^6 steps, we simulate the shift in filament composition from the Wide Helix to the Tight Helix, by activating the Tight Helix to let it slowly constrict. For this we have deleted the rigid bonds between the Wide Helix and the Tight Helix and let them move independently. The Wide Helix remains in its previous state with a target radius of 30.8 nm. Meanwhile the Tight Helix starts off in exactly the same state as the Wide Helix, but progressively reduces its target radius from 30.8 nm to around 3 nm, which takes 2.5×10^6 steps in total. Half way through this stage, we instantaneously disassemble the Wide Helix, to prevent the Tight Helix from detaching from the membrane. In a final step, the Tight Helix disassembles, leading to the release of the cargo-containing vesicle.

To test the effect of cargo size on scission, we repeat the same simulation with different cargo sizes $r_{\text{cargo}} = \{1\sigma, 4\sigma, 6\sigma, 10\sigma\}$. The associated membrane-cargo interaction strength are $\{12k_\text{B}T$, $1.8k_\text{B}T$, $0.8k_\text{B}T$, $0.5k_\text{B}T\}$, respectively.

To test the effect of membrane bending rigidity on the membrane remodelling events, we repeat the same simulation with $\mu = 5$ in the membrane model, which yields a more rigid membrane with rigidity $\sim 40k_\text{B}T$.

When the cargo is absent, the same simulation protocol as described in the section above is adopted, except that we do not include the cargo particle.

S2.3 Simulation of co-equilibration of the Spiral and Wide Helix

To prepare the system, both filaments are initialized as loosely-packed spirals in the flat state, with each filament constructed of 186 subunits. A layer of 7360 membrane beads ($80\times80\sigma^2$) are positioned under the filaments in the x-y plane. Note that this initial configuration and part of the simulation described in this section are reused from a previous study [6], where the Helix is the outer strand, yet this should not change our conclusions on copolymer rigidity and stability. Initially, the membrane is equilibrated for 10^4 steps

while the protein filaments are kept frozen. The protein filaments are then released with both filaments in the flat state for about 10^6 steps.

For the study of filament rigidity (Fig. 2 A), target radii for the Spiral and the Helix are kept constant at $R_1 = 13.2$ nm, $R_2 = 15.8$ nm, respectively, while we vary the bond strengths of the two filaments and run each simulation for around 10^6 steps. Typically, a stable deformation is formed within 10^5 steps. Finally, the Spiral is disassembled and only the Helix is kept on the membrane for another 10^6 steps.

To test the stability of the copolymer scaffold (Fig. 2 C), we keep the target radius of both strands at $R_1 = 19.4$ nm initially and instantaneously change the target geometry of the Helix to the tilted state at different target radii R_2 for different simulations. After around 10^6 steps of each simulation, we check whether both filaments remain attached to the membrane.

S2.4 Simulation of co-equilibration of the Wide Helix and Tight Helix

To test the stability of the copolymer scaffold (Fig. S3), we restart from one snapshot from the cargo-containing simulations, when the Wide Helix and the Tight Helix form a copolymer. The target radii of both helices are initially set to different R_2 and equilibrated for 10^6 steps. Then the target radius of the Tight Helix is instantaneously decreased to $R_3 = 5.3$ nm and we let the system equilibrate for 10^5 steps, before checking whether both filaments remain attached to the membrane.

S2.5 Simulation of the Tight Helix constriction and disassembly

To prepare the system, we place a single ESCRT-III filament on the membrane (16560 membrane beads, $120 \times 120\sigma^2$) with the cargo particle in its middle, and carry out the simulation with the filament in the flat state for 2×10^4 steps. We then switch the filament to a tilted state of target radius $R_{\text{init}} = 17$ nm and run the simulation for $\sim 2 \times 10^6$ steps. We simulate the filament constriction by progressively reducing its target radius from R_{init} to R_{final} at a constant rate of constriction $r_{\text{constriction}}$. The rate of constriction is defined as $r_{\text{constriction}} = (R_3[t] - R_3[t + 10\tau])/10\tau$ for any time t, where R_3 is the target radius of the Tight Helix, τ is the MD time unit. The filament is disassembled after constriction and four protocols for disassembly are tried (i.e., "instantaneous", "random", "from top", "from bottom").

The scission appears to be robust against cargo leakage. As a sanity check, we substitute the single generic cargo (radius $r = 8\sigma$) with six small cargos ($r = 2\sigma$), once the neck is thin enough to be able to sterically confine the cargos inside the budding vesicle. Volume-exclusion interactions are applied between the cargo particles (interaction strength $\epsilon = 2k_{\text{B}}T$) and short-ranged L-J interactions are applied between the cargo particles and the membrane beads (interaction strength $\epsilon = 5k_{\text{B}}T$). We carry out five replica simulations and find that four of them are successful in scission (see typical trajectory in Fig. S6). In the unsuccessful scission trajectory, the membrane retracts after disassembly of the Tight Helix filament and the cargos are not leaked out either.

S2.6 Mapping time unit from MD unit (τ) to physical unit (μs)

The mapping expression between physical and simulation units of time is obtained by comparing the diffusion constant measured in our computer model to experimental values. To this end, we take a layer of 29440 membrane beads ($160 \times 160\sigma^2$) and equilibrate the system for 10^4 steps with the same MD setup as detailed in the previous paragraphs. We then run the equilibration trajectory for another 3×10^5 steps and measure the mean squared displacement (MSD) of a membrane bead in the 2D membrane layer. Using the relation D= MSD/$4t$, we obtain the in-plane diffusion constant of the membrane bead D= $0.06\sigma^2/\tau$. Mapping our computed diffusion constant to the typical value for the diffusion constant of a lipid molecule in a bilayer membrane ($20\mu m^2/s$ [8]) gives our MD time unit $\tau \sim 0.018\mu s$. A similar timescale is obtained when mapping the diffusion constant of a freely-moving three-beaded protein unit in 3D (D= MSD/$6t = 0.25\sigma^2/\tau$) to the diffusion constant of small soluble proteins (D=$100\mu m^2/s$ [9]), which gives $\tau \sim 0.013\mu s$. However, note that one bead in our model represents a patch of lipids rather than a single lipid molecule, and our three-beaded

protein unit does not necessarily represent a single ESCRT protein but rather a segment of the ESCRT polymer. Therefore, the timescale obtained above ($\tau = 0.02\mu s$) only serves as a lower bound.

References for S2 Appendix

[1] Harker-Kirschneck L, Baum B, Šarić A. Changes in ESCRT-III filament geometry drive membrane remodelling and fission in silico. BMC Biology. 2019;doi:10.1186/s12915-019-0700-2.

[2] Yuan H, Huang C, Li J, Lykotrafitis G, Zhang S. One-particle-thick, solvent-free, coarse-grained model for biological and biomimetic fluid membranes. Phys Rev E Stat Nonlin Soft Matter Phys. 2010;82(1). doi:10.1103/PhysRevE.82.011905.

[3] Henne WM, Buchkovich NJ, Zhao Y, Emr SD. The endosomal sorting complex ESCRT-II mediates the assembly and architecture of ESCRT-III helices. Cell. 2012;doi:10.1016/j.cell.2012.08.039.

[4] Shen QT, Schuh AL, Zheng Y, Quinney K, Wang L, Hanna M, et al. Structural analysis and modeling reveals new mechanisms governing ESCRT-III spiral filament assembly. J Cell Bio. 2014;doi:10.1083/jcb.201403108.

[5] Kozlovsky Y, Kozlov MM. Stalk model of membrane fusion: Solution of energy crisis. Biophys J. 2002;82(2). doi:10.1016/S0006-3495(02)75450-7.

[6] Pfitzner AK, Mercier V, Jiang X, Moser von Filseck J, Baum B, Šarić A, et al. An ESCRT-III Polymerization Sequence Drives Membrane Deformation and Fission. Cell. 2020;doi:10.1016/j.cell.2020.07.021.

[7] Plimpton S. Fast parallel algorithms for short-range molecular dynamics. J Comput Phys. 1995;117(1). doi:10.1006/jcph.1995.1039.

[8] Fahey PF, Webb WW. Lateral diffusion in phospholipid bilayer membranes and multilamellar liquid crystals. Biochemistry. 1978;17(15):3046. doi:doi: 10.1021/bi00608a016.

[9] Young ME, Carroad PA, Bell RL. Estimation of diffusion coefficients of proteins. Biotechnol Bioeng. 2010;22(5):947–955. doi:10.1002/bit.260220504.
