# Supplementary material for: Modelling membrane reshaping by staged polymerization of ESCRT-III filaments
Source: PLoS Comput Biol. 2022 Oct 17;18(10):e1010586. doi: 10.1371/journal.pcbi.1010586 (PMC9612822; doi:10.1371/journal.pcbi.1010586)
Supplement: S3 Appendix — (PDF) [file pcbi.1010586.s003.pdf]

S3 Analysis Protocols

S3.1 Characterization of buckle deformation

The membrane deformation is characterized by the semi-cone angle θ, which is defined as the angle between the vertical axis (normal to the original flat membrane plane) and the membrane normal at half depth of the deformation. For the one-bead-thick membrane model, we take the dipole directions of the beads as the norm directions. The snapshots are saved every 1000 steps and the last 200 snapshots of the simulation are used to calculate the thermodynamic average for each simulation. The numerical values are averaged over five simulations with independent velocity seeds.

S3.2 Measurement of neck radius

Throughout this work, the neck radius is defined as the distance from the middle of the neck, to the inner surface of the membrane beads. To obtain it, we first measure the membrane neck radius from the centre of the neck to centre of the membrane beads. The snapshots are saved every 10,000 steps along the constriction trajectories. For each snapshot, we first extract the centre of mass of the Tight Helix in the vertical direction, z_{TH}. The neck is then sliced into rings of thickness σ and the diameter of each ring is estimated by the maximal distance between any two membrane beads within this ring. The neck radius is then estimated by averaging over half of the diameter of these rings that are located within $z_{\mathrm{TH}} \pm 2\sigma$. Finally, 0.5σ is deducted from the raw neck radius to obtain the inner surface membrane neck radius.

S3.3 Calculation of membrane potential energy profile

The bending and stretching energy between each pair of membrane beads, the membrane-filament adhesion energy between a membrane bead and a filament bottom bead, and the membrane-cargo adhesion energy between a membrane bead and the cargo bead are computed between each pair of beads in the system and evenly distributed between these two beads. The total potential energy of each membrane bead is obtained by summing up all of its pair interaction energies with all other beads. The three decomposed membrane potential energy profiles (i.e., bending and stretching, membrane-filament, membrane-cargo) are obtained by summing up only specific type of pair-pair interactions between each membrane bead and all other beads. The potential energy profiles are then binned along the membrane neck, using the bin size σ and averaged over eight to ten snapshots after binning. Finally, the total potential energies shown in Fig. 3C are applied a constant shift so that the flat membrane has energy around zero. For visualization purposes, surface mesh is constructed by Ovito [1] using Gaussian density method [2] with resolution = 400 and iso value = 0.7.

S3.4 Quantification of scission efficiency

Membrane scission is detected by a DBSCAN clustering algorithm (eps=1.4, min_samples=1) since the membrane undergoes a topological transition from one cluster to two during the scission event. To quantify the scission efficiency, we carry out 50 to 60 independent constriction and disassembly simulations. Ten independent simulations are used to define a measurement and the scission efficiency is calculated by counting the number of successful scission trajectories out of those ten. We then obtain the scission efficiency by averaging over five to six independent measurements.

References for S3 Appendix

[1] Stukowski A. Visualization and analysis of atomistic simulation data with OVITO-the Open Visualization Tool. Model Simul Mat Sci Eng. 2010;18(1). doi:10.1088/0965-0393/18/1/015012.

[2] Krone M, Stone J, Ertl T, Schulten K. Fast visualization of Gaussian density surfaces for molecular dynamics and particle system trajectories. EuroVis-Short Papers. 2012;doi:10.2312/PE/EuroVisShort/EuroVisShort2012/067-071.
