# Supplementary material for: Modelling membrane reshaping by staged polymerization of ESCRT-III filaments
Source: PLoS Comput Biol. 2022 Oct 17;18(10):e1010586. doi: 10.1371/journal.pcbi.1010586 (PMC9612822; doi:10.1371/journal.pcbi.1010586)
Supplement: S1 Table — (PDF) [file pcbi.1010586.s004.pdf]

Table S1: Summary of all bead-bead interactions (A-B) and their default interaction strength.

| **Type of bead A** | **Type of bead B** | **Interaction Type** | **Interaction strength** |
|---|---|---|---|
| Membrane beads | membrane beads | Yuan et al. [1] | $\epsilon = 4.34 k_{\mathrm{B}} T$ |
| Protein beads from adjacent subunits in the same filament | | harmonic bonds | $k = 256 k_{\mathrm{B}} T / \sigma^2$ |
| Beads within the same three-bead protein subunit | | rigid body | NA |
| Top beads in the three-bead protein subunit | protein beads | volume exclusion | $\epsilon = 2.0 k_{\mathrm{B}} T$ |
| Top beads in the three-bead protein subunit | membrane beads | volume exclusion | $\epsilon = 2.0 k_{\mathrm{B}} T$ |
| Bottom beads in the three-bead protein subunit | membrane beads | Lennard Jones | $\epsilon = 3.0 k_{\mathrm{B}} T$ |
| Protein bottom beads at the interfaces of different filaments | | Lennard Jones | $\epsilon = 3.0 k_{\mathrm{B}} T$ |
| Protein bottom beads that are not at the filament interfaces | | volume exclusion | $\epsilon = 2.0 k_{\mathrm{B}} T$ |
| Cargo bead | protein beads | volume exclusion | $\epsilon = 2.0 k_{\mathrm{B}} T$ |
| Cargo bead | membrane beads | Lennard Jones | $\epsilon = 0.6 k_{\mathrm{B}} T$ |

## Reference


[1] Yuan H, Huang C, Li J, Lykotrafitis G, Zhang S. One-particle-thick, solvent-free, coarse-grained model for biological and biomimetic fluid membranes. Phys Rev E Stat Nonlin Soft Matter Phys. 2010;82(1). doi:10.1103/PhysRevE.82.011905.
