# Supplementary material for: Modelling membrane reshaping by staged polymerization of ESCRT-III filaments
Source: PLoS Comput Biol. 2022 Oct 17;18(10):e1010586. doi: 10.1371/journal.pcbi.1010586 (PMC9612822; doi:10.1371/journal.pcbi.1010586)
Supplement: S2 Table — (PDF) [file pcbi.1010586.s005.pdf]

Table S2: Conversion table to map values from MD units to physical units.

| quantity | MD unit | physical unit |
|---|---|---|
| length | $\sigma$ | 2.3 nm [a] |
| time | $\tau$ | 0.02 $\mu s$ |
| energy | $k_{\mathrm{B}}T$ | 0.6 kcal/mol [b] |

[a] Taken from Ref. [1]
[b] Assuming $T$=300K.

# Reference


[1] Harker-Kirschneck L, Baum B, Šarić A. Changes in ESCRT-III filament geometry drive membrane remodelling and fission in silico. BMC Biology. 2019;doi:10.1186/s12915-019-0700-2.
