# Supplementary material for: Modelling membrane reshaping by staged polymerization of ESCRT-III filaments
Source: PLoS Comput Biol. 2022 Oct 17;18(10):e1010586. doi: 10.1371/journal.pcbi.1010586 (PMC9612822; doi:10.1371/journal.pcbi.1010586)
Supplement: S1 Fig — (PDF) [file pcbi.1010586.s006.pdf]

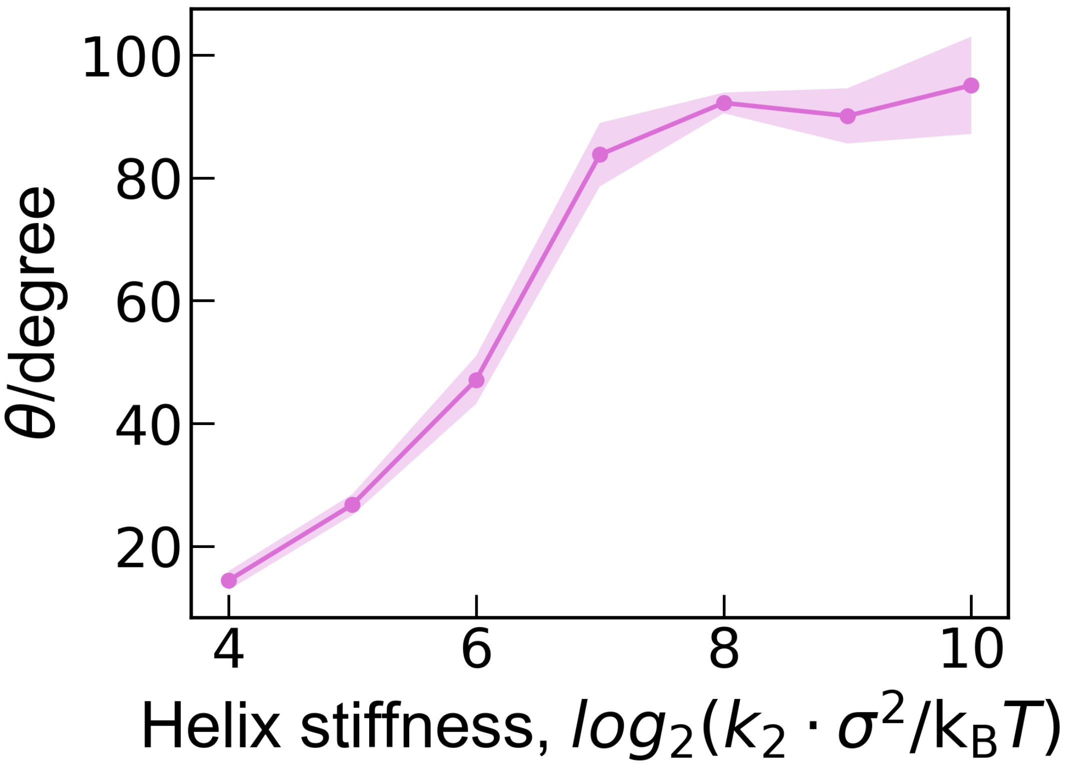

Figure S1: Membrane deformation as a function of bond stiffness of the Wide Helix (k_2). The membrane deformation angle θ is averaged over 5 independent simulations with the standard deviation indicated by the shaded area.
