# Supplementary material for: Modelling membrane reshaping by staged polymerization of ESCRT-III filaments
Source: PLoS Comput Biol. 2022 Oct 17;18(10):e1010586. doi: 10.1371/journal.pcbi.1010586 (PMC9612822; doi:10.1371/journal.pcbi.1010586)
Supplement: S2 Fig — (PDF) [file pcbi.1010586.s007.pdf]

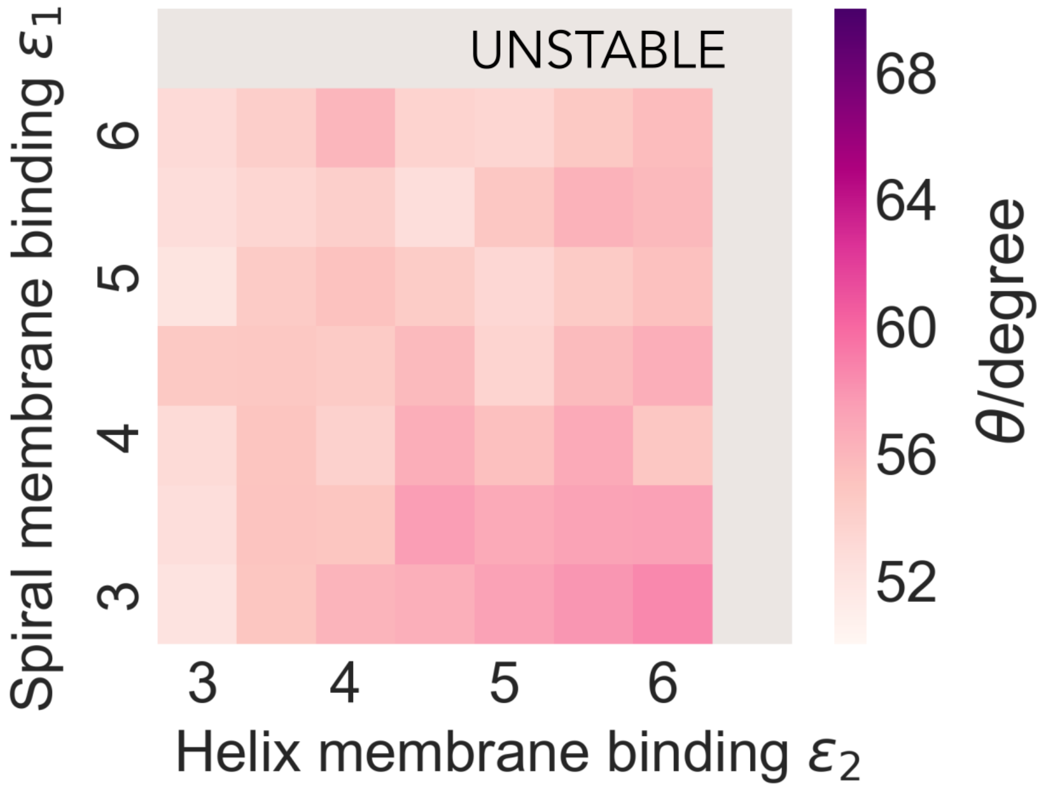

Figure S2: Membrane deformation as a function of membrane-binding affinity of the Spiral (ϵ_1) and the Helix (ϵ_2), (ϵ_1, ϵ_2 are in the units of $\mathrm{k_B}T$). Filament stiffness is set as $k_1 = k_2 = 256\ \sigma^2/\mathrm{k_B}T$. The membrane deformation is characterized by the angle between the vertical and the membrane norm at half depth of the deformation, as illustrated in Fig 2B. The membrane deformation is averaged over 5 independent simulations.
