# Supplementary material for: Modelling membrane reshaping by staged polymerization of ESCRT-III filaments
Source: PLoS Comput Biol. 2022 Oct 17;18(10):e1010586. doi: 10.1371/journal.pcbi.1010586 (PMC9612822; doi:10.1371/journal.pcbi.1010586)
Supplement: S3 Fig — (PDF) [file pcbi.1010586.s008.pdf]

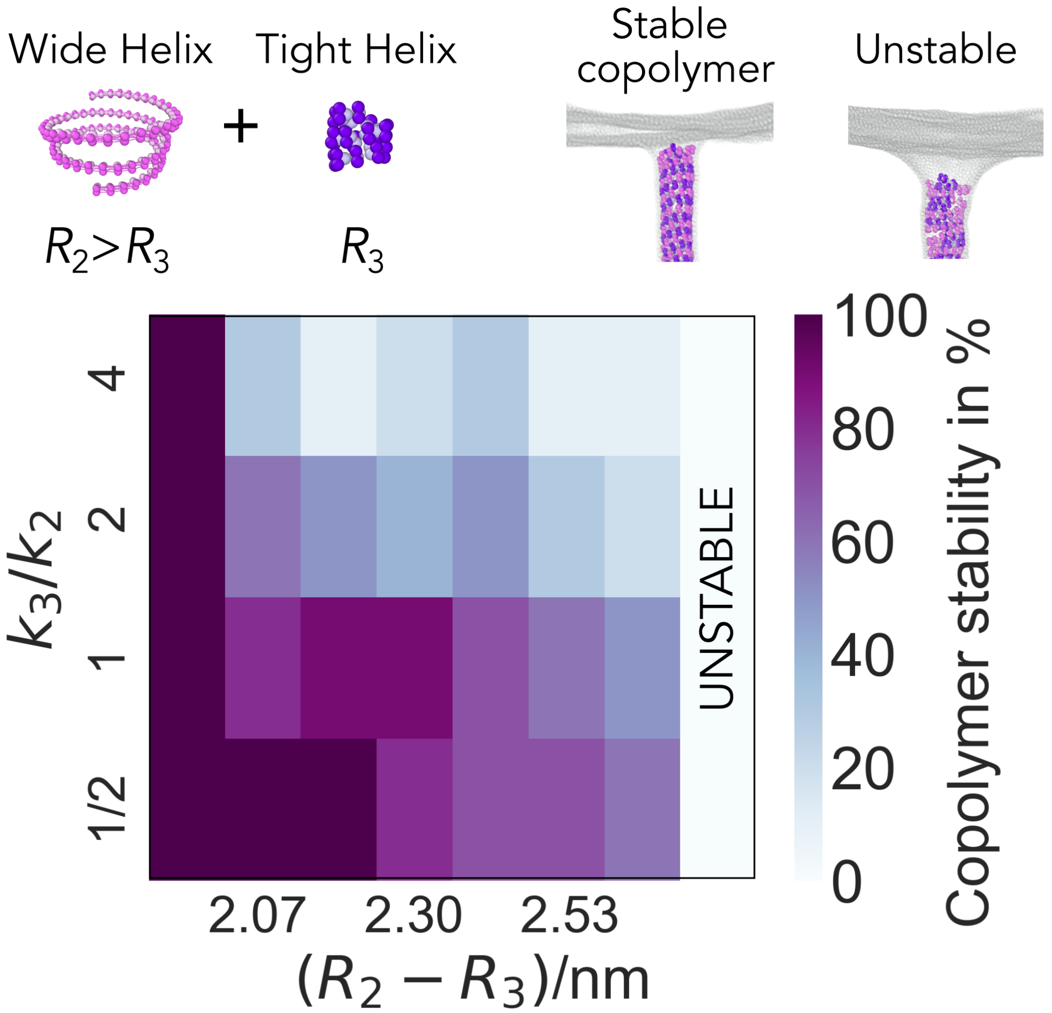


Figure S3: Copolymer stability as a function of the mismatch in the radius and ratio of stiffness between the Tight Helix and the Wide Helix. All data points are averaged over ten simulations. The Tight Helix is fixed at radius $R_3 = 5.3$ nm with filament bond stiffness $k_3 = 256\ \mathrm{k_B}T/\sigma^2$.
