# Supplementary material for: Modelling membrane reshaping by staged polymerization of ESCRT-III filaments
Source: PLoS Comput Biol. 2022 Oct 17;18(10):e1010586. doi: 10.1371/journal.pcbi.1010586 (PMC9612822; doi:10.1371/journal.pcbi.1010586)
Supplement: S4 Fig — (PDF) [file pcbi.1010586.s009.pdf]

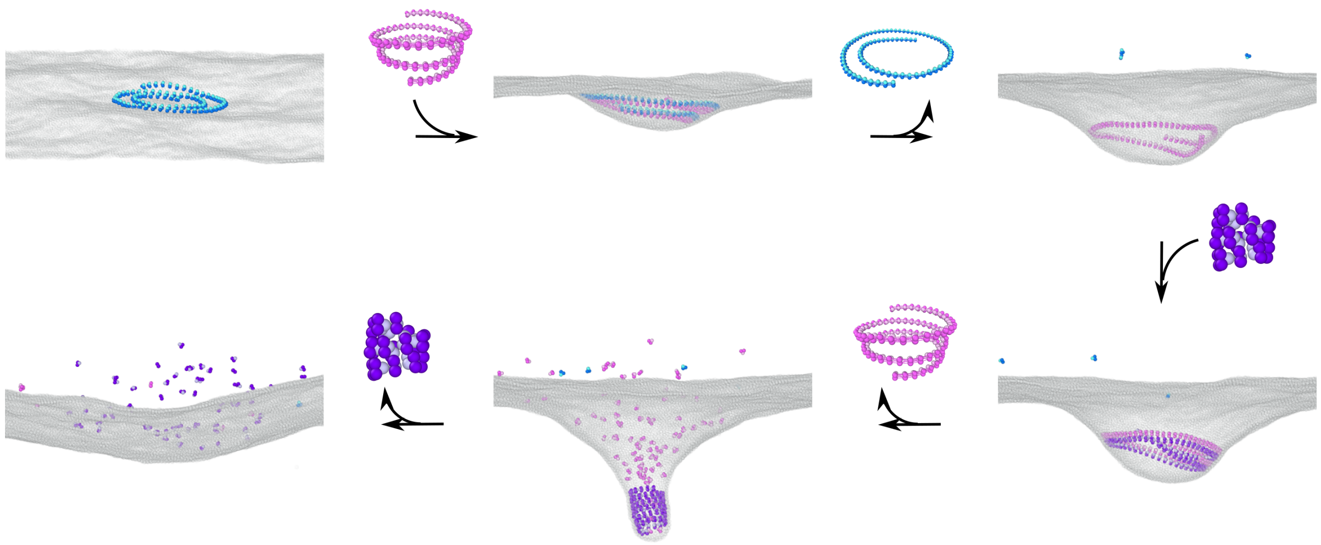

Figure S4: Typical snapshots along the trajectory where the three filaments are activated and disassembled in a stepwise manner in the absence of cargo.
