# Supplementary material for: Modelling membrane reshaping by staged polymerization of ESCRT-III filaments
Source: PLoS Comput Biol. 2022 Oct 17;18(10):e1010586. doi: 10.1371/journal.pcbi.1010586 (PMC9612822; doi:10.1371/journal.pcbi.1010586)
Supplement: S5 Fig — (PDF) [file pcbi.1010586.s010.pdf]

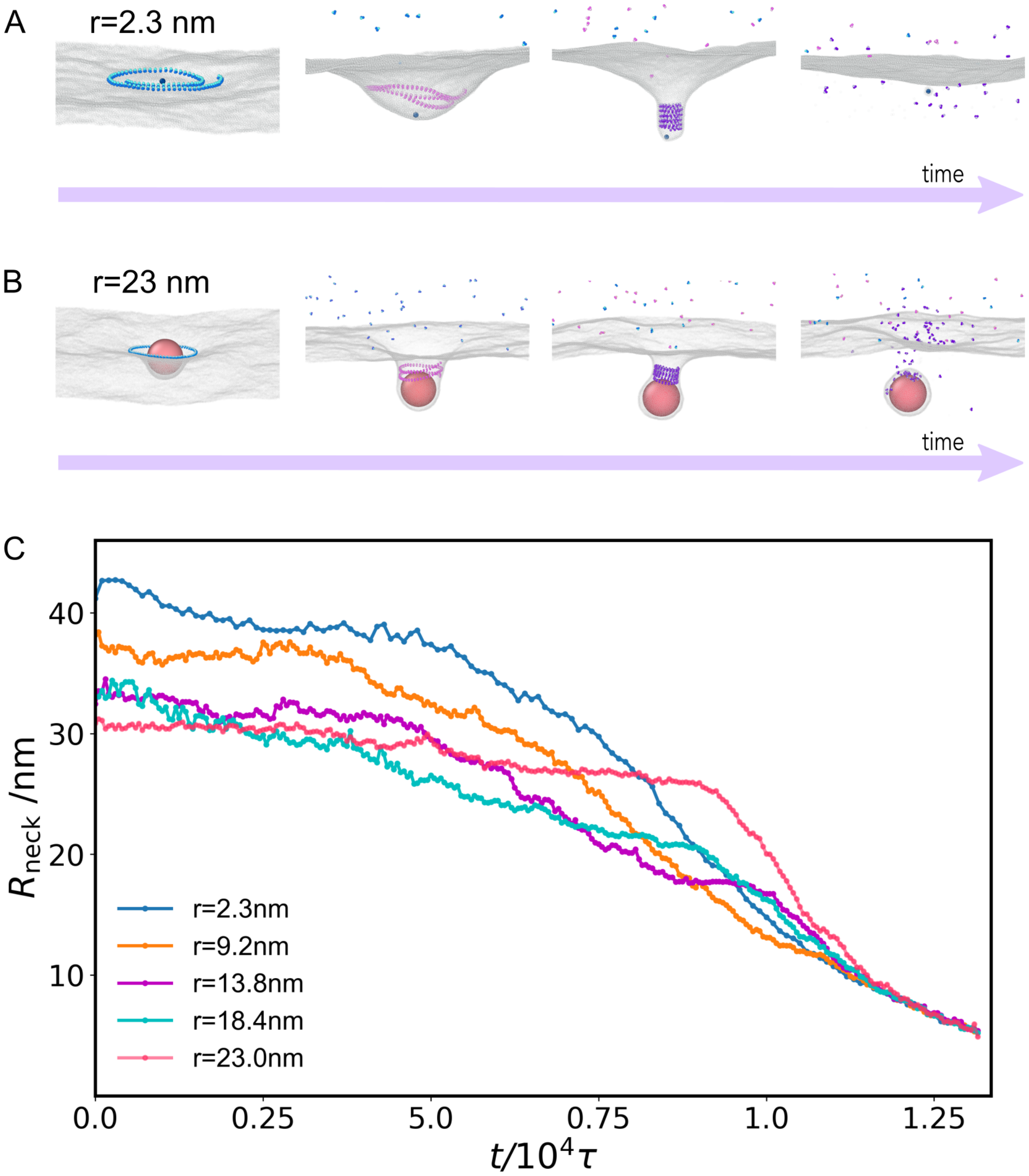

Figure S5: Impact of cargo size on the sequential recruitment and disassembly of different ESCRT-III filaments. Panels A, B: Snapshots along the trajectories of the system where the three filaments are activated and disassembled in a stepwise manner, with the radius of the generic cargo set as 2.3 nm (A) and 23 nm (B), respectively. C: Neck radii along the constriction trajectories for different cargo sizes.
