# Supplementary material for: Modelling membrane reshaping by staged polymerization of ESCRT-III filaments
Source: PLoS Comput Biol. 2022 Oct 17;18(10):e1010586. doi: 10.1371/journal.pcbi.1010586 (PMC9612822; doi:10.1371/journal.pcbi.1010586)
Supplement: S6 Fig — (PDF) [file pcbi.1010586.s011.pdf]

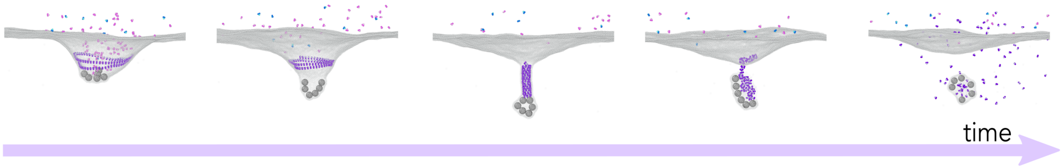

Figure S6: Tight Helix constriction and scission with six smaller volume-excluded cargos, rather than one single large volume-excluded cargo. Cargo radius $r_{\text{cargo}} = 2\sigma$.
