# Supplementary material for: Modelling membrane reshaping by staged polymerization of ESCRT-III filaments
Source: PLoS Comput Biol. 2022 Oct 17;18(10):e1010586. doi: 10.1371/journal.pcbi.1010586 (PMC9612822; doi:10.1371/journal.pcbi.1010586)
Supplement: S7 Fig — (PDF) [file pcbi.1010586.s012.pdf]

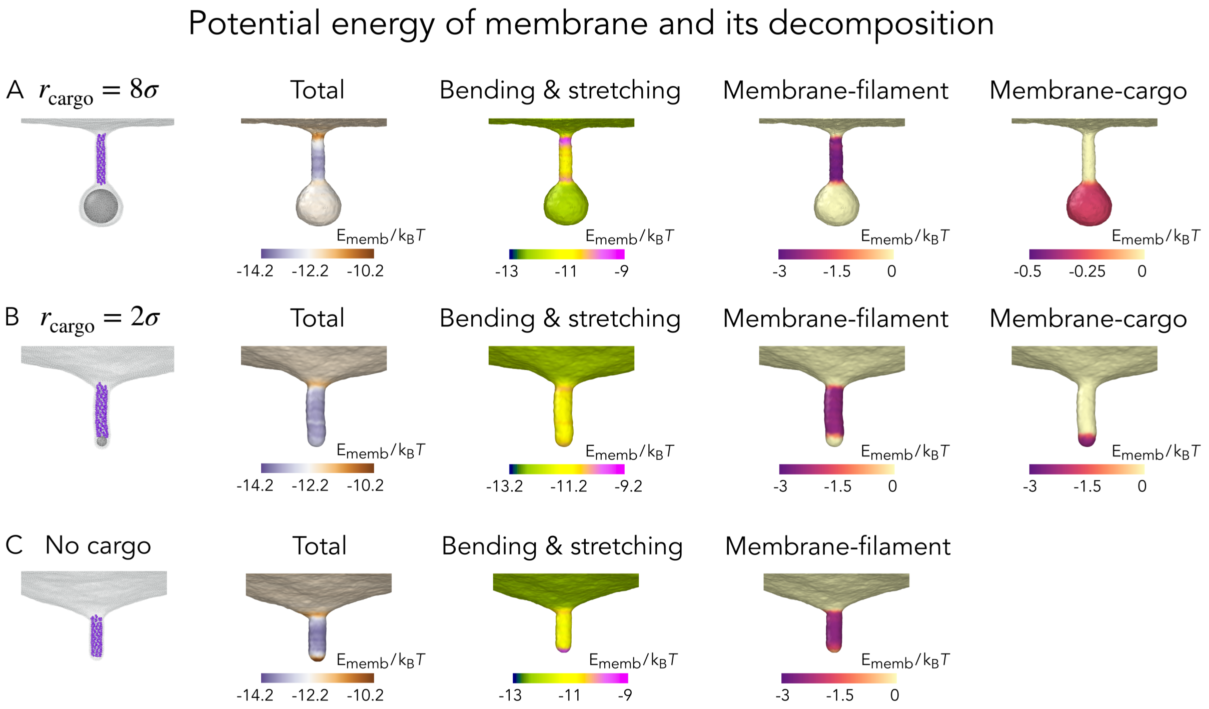

Figure S7: The local pair potential energy (i.e., energy per bead) of the membrane computed on representative snapshots before membrane breakage in the presence of the generic cargo with radii $r_{cargo} = 8\sigma$ (A), $r_{cargo} = 2\sigma$ (B), and in the absence of the generic cargo (C). The total potential is the sum of the following three potential energy terms, which are plotted separately: bending and stretching mechanical energies computed from Yuan et al. [1], filament-membrane and cargo-membrane adhesion energies computed from short-ranged LJ potential. The energies are binned along the neck of the tube with bin width σ and averaged over 10 snapshots.

Reference

[1] Yuan H, Huang C, Li J, Lykotrafitis G, Zhang S. One-particle-thick, solvent-free, coarse-grained model for biological and biomimetic fluid membranes. Phys Rev E Stat Nonlin Soft Matter Phys. 2010;82(1). doi:10.1103/PhysRevE.82.011905.
