# Supplementary material for: Modelling membrane reshaping by staged polymerization of ESCRT-III filaments
Source: PLoS Comput Biol. 2022 Oct 17;18(10):e1010586. doi: 10.1371/journal.pcbi.1010586 (PMC9612822; doi:10.1371/journal.pcbi.1010586)
Supplement: S8 Fig — (PDF) [file pcbi.1010586.s013.pdf]

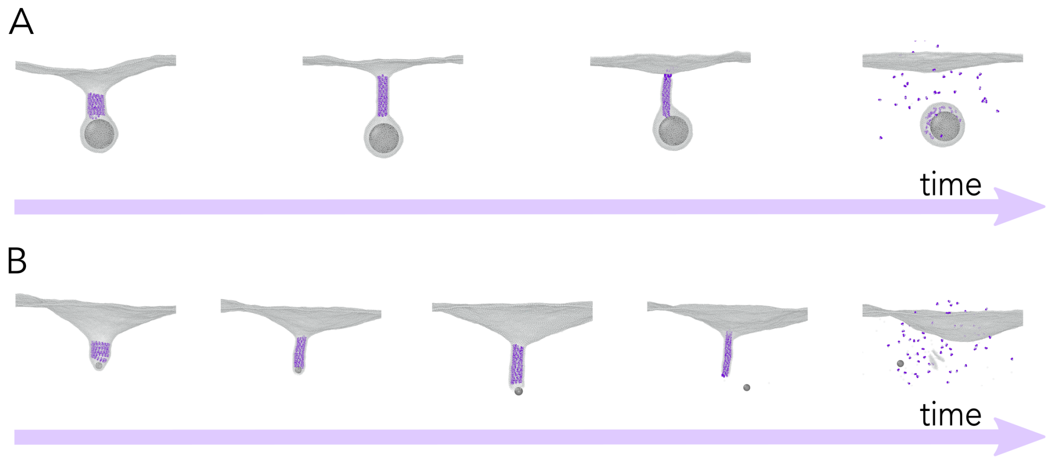

Figure S8: Tight Helix constriction and scission when the cargo-membrane adhesion is replaced by pure volume-exclusion. This change in interaction is applied once the membrane neck is thin enough to be able to sterically confine the cargo particle inside the budding vesicle or at the tip of the invagination. A: Cargo size $r_{\text{cargo}} = 8\sigma$. Successful scission is achieved with membrane breakage at the top rim of the neck. B: Cargo size $r_{\text{cargo}} = 2\sigma$. Membrane breaks at the bottom tip of the membrane neck, resulting in cargo leakage and membrane retraction.
