# Supplementary material for: Modelling membrane reshaping by staged polymerization of ESCRT-III filaments
Source: PLoS Comput Biol. 2022 Oct 17;18(10):e1010586. doi: 10.1371/journal.pcbi.1010586 (PMC9612822; doi:10.1371/journal.pcbi.1010586)
Supplement: S9 Fig — (PDF) [file pcbi.1010586.s014.pdf]

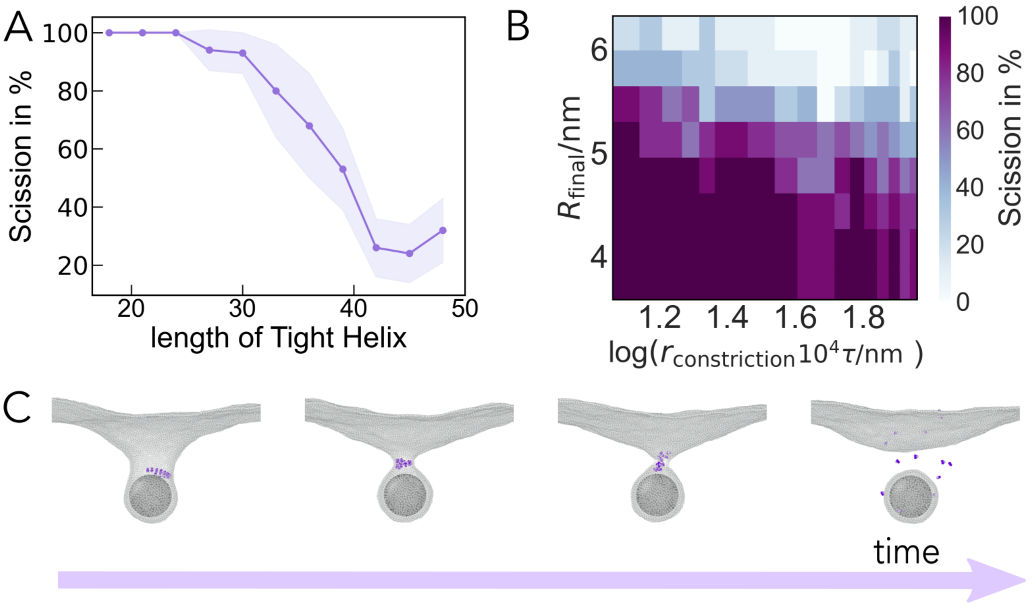

Figure S9: Shortening the length of the Tight Helix promotes scission. A: Scission efficiency as a function of the length of the Tight Helix (in number of monomers) following the protocol of progressive Tight Helix constriction followed by instantaneous disassembly. All the data is collected for $r_{\text{constriction}} = 2.1 \times 10^{-3}\text{nm}/\tau$ and $R_{\text{final}} = 4.3$ nm. The data is averaged over 5 independent measurements with the standard deviation indicated by the shaded area. B: Scission efficiency as a function of the final target radius of the Tight Helix containing 13 monomers, R_{final}, and the rate of the Tight Helix constriction, $r_{\text{constriction}}$. Each data point is computed from 10 independent simulations. C: Representative snapshots along the scission trajectory of the 13-monomer Tight Helix. R_{final}=5.6 nm and $r_{\text{constriction}} = 9.2 \times 10^{-3}\text{nm}/\tau$.
