# Supplementary material for: Modelling membrane reshaping by staged polymerization of ESCRT-III filaments
Source: PLoS Comput Biol. 2022 Oct 17;18(10):e1010586. doi: 10.1371/journal.pcbi.1010586 (PMC9612822; doi:10.1371/journal.pcbi.1010586)
Supplement: Attachment — Submitted filename: reply-letter.pdf [file pcbi.1010586.s021.pdf]

Dear Prof. Gov,

Thank you for taking the time to review our manuscript. We are very grateful to the two reviewers for their interest in our work and their excellent comments and suggestions, which have helped us improve the paper. To address the reviewer's comments, we have performed new simulations and have refined the text to make our procedure and findings clearer. In particular, we have now:

- Carried out a more detailed analysis of the membrane energy during deformation, accompanied by a set of new simulations. These analyses predict the location of the membrane breakage
- Carried out extra simulations using a shorter Tight Helix, to test how filament length influences scission efficiency with our molecular dynamics approach
- Repeated some of the simulations with a higher membrane bending rigidity at the upper limit of the biological range, as a sanity check
- Repeated the scission trajectory using multiple smaller cargos instead of a single larger cargo particle. This shows scission can occur without leaks even for small cargos.
- Added Table S1 and Table S2 to better organize the presentation of our simulation setup

We have also addressed all the reviewers' comments in the revised version of the manuscript, as detailed below. A copy of the main text and the SI with changes highlighted was submitted as supporting material. We hope that you now deem our paper suitable for publication in PLOS Computational Biology.

Sincerely,

Xiuyun Jiang, Lena Harker-Kirschneck, Christian Vanhille-Campos, Anna-Katharina Pfitzner, Elene Lominadze, Aurélien Roux, Buzz Baum, and Anđela Šarić

Reviewer #1:

- Based on the results of the model, could the authors suggest some experiments to validate some of the predictions of the model? I feel this could help experimental biophysicists see the relevance of the findings herein presented.

This is a nice suggestion and some of our findings are indeed experimentally testable.

Our predictions on the influence of the rate of filament constriction and disassembly on the scission process could be tested using mutants that alter Vps4 activity and/or the binding of Vps4 to target ESCRT-III subunits. Our model predicts a relationship between the protocol of filament disassembly and the partitioning of disassembled ESCRT monomers (i.e., "ESCRT in bud" in Fig.4C) which we would hope could be tested by a more thorough experimental quantification of the amount of ESCRT-III proteins in vesicles. We have added a comment on this in the discussion section.

- The section named "Model for sequential recruitment..." is generally clear and well written, but I think it could help the readers to present tighter connections with the experiments (especially in vitro experiments). For example, in the model only 3 filaments are included. Is there something known whether 3 filaments (possibly formed by the 5 proteins mentioned by the authors) are enough to drive membrane remodelling?

We thank the reviewer for this important comment. Indeed, there is experimental knowledge on ESCRT-III filament constitution which supports our three-filament minimal model. Taking yeast ESCRT-III as an example, extensive *in vitro* studies suggest that, Vps24 always polymerizes together with Vps2 to form heteropolymers (Babst et al. 2002a; Lata et al. 2008; Teis et al. 2008; Mierzwa2017; Pfitzner2020; Banjade et al. 2021). Cryo-EM structure of the Did2-Ist1 complex suggests that Ist1 does not bind to and polymerize directly on the membrane, but instead exerts its reshaping force on the membrane indirectly by forming a second strand on top of the Did2 strand (McCollough et al. 2015; Nguyen et al. 2020). Therefore, not every ESCRT isoform forms a separate strand. This influenced our choice of a three (hetero) polymer-model, which we show here can induce scission. We have now included a comment on this at the end of the first paragraph, model section to discuss this choice. As detailed below, it is also possible that in some instances two filaments may suffice, especially if the initial binding event is aided by other machinery, e.g. ESCRT-II. In line with this, many archaeal genomes possess only two ESCRT-III proteins, a single Vps4, and ESCRT-I and II complexes.

- The authors use a bilayer with a bending rigidity of 15 kBT. This is fine, probably in the low range of biological bilayers. ESCRTs however act at different membranes within the cell, which

could/might have different rigidities (up to ~40 kBT possibly). Have the authors tested whether the actual value of kappa (within a factor of 2) has any significant role in the observed effects?

We have repeated the whole vesicle budding simulation (with cargo) using a different bending rigidity-related parameter in the membrane potential ($\mu = 5$ instead of $\mu = 3$, as per the original potential annotation), which according to the original paper yields a bending rigidity of $\kappa = 40\ k_B T$. We do not see any qualitative difference in the sense that conical membrane deformation and fission are both successful using the same filament bond stiffness as in the original manuscript. We have also uploaded this trajectory as Video 2 and commented on it at the beginning of the results section.

Please note that upon revising our parameters, we noticed that the bending rigidity we used was $\sim 20\ k_B T$ instead of $\sim 15\ k_B T$, which we have now corrected in the manuscript.

- The fission pores observed in the simulations, are always non-leaky, or did the authors observed in some cases leaky fission?

The amount of ESCRT protein leakage in our model depends on the detailed order of disassembly (as can be seen in Fig.4A snapshots), and some protocols lead to more ESCRT leakage than others. However, it is important to add that our coarse-grained model, just like any continuum model, does not necessarily capture the fine details of the membrane fission and fusion processes at scales below ~10nm, as it lacks the explicit bilayer structure. It is hence possible that membrane pores in our model are longer-lived than they would be in an explicit bilayer, which would lead to an overestimation of ESCRT leakage in our model.

In terms of the cargo leakage, which is a more important aspect, we have never observed the leakage of the single generic cargo. As the breakage of membrane neck always occurs at its top rim, we do not expect the leakage to occur even for a collection of smaller cargos. Nevertheless, we have rerun the filament constriction and scission simulation with 6 smaller cargos ($r_{cargo} = 2\sigma$) which are volume-excluded to each other, to investigate the robustness of our model with respect to multiple non-cohesive transporting materials. We find this scenario also to be successful in scission (80% efficiency). As expected, the neck still breaks at the top and we do not observe any cargo leakage. Regarding the unsuccessful scission trajectory, the membrane retracted after Tight Helix disassembly and the cargos did not leak out either.

We have now commented on cargo leakage in section “Membrane scission is sensitive to constriction kinetics” on page 5 and added Fig.S6 to show one such trajectory.

- When the authors say (pg. 5, last paragraph) that scission always occurs where the energy is highest, is that because in the model, the interaction of the cargo with the membrane is large enough to stabilize the bottom part of the tube? What would happen if the cargo is only anchored through a point (e.g. to the tip of the invagination?).

This is an excellent point, which encouraged us to revisit this phenomenon and we believe that we now show a better analysis of the location of membrane breakage.

We have carried out two control sets of scission simulations, in which, once the tight neck is formed i) the cargo is anchored to the membrane only at one point via a harmonic bond ii) the cargo-membrane interaction is changed from attractive to volume exclusion only.

Interestingly, for cargo size r=8σ, which would correspond to a cargo of diameter ~40 nm, on the order of biologically relevant bud sizes, the membrane still breaks at the top of the membrane neck in both sets of simulations. However, for very small cargo size r=2σ, the membrane neck breaks at the bottom tip, just like in the “no-cargo” simulations, and the cargo leaks out without forming a bud. Therefore, we conclude that for large cargo sizes where both top and bottom of the neck rims are curved in the saddle shape, the higher local membrane bending at the top rim of the neck is sufficient to trigger the initial membrane breakage. But for very small cargo where the tubular deformation is capped by a conical tip, the cargo-membrane interaction is needed to avoid the tip breakage and allow for bud formation. Although the small-cargo scenario is less biologically relevant, it allows us to have a better understanding of the role that the cargo plays in mediating scission. This may also point to roles for ESCRT-I and II complexes in the process – something we would hope to consider in future work.

We have updated Fig.3C to plot the total potential energy of the membrane, which now includes filament binding energy, cargo-membrane adhesion energy, as well as the bending and stretching energies, added Fig. S7 to show all the separate energy contributions for r=8σ and r=2σ cargos, and added Fig. S8 to show that scission is successful for r=8σ cargo but fails for r=2σ cargo when we have volume exclusion instead of L-J interactions between the cargo and the membrane. These extra data indicate that cargo plays an important role in mediating scission by curvature generation and, for small cargos, membrane stabilization. We have included a comment on this in the section “Membrane scission is sensitive to constriction kinetics” at the first paragraph of page 6 and in the second-to-last paragraph of the discussion section.

- In the methods (pg 9, last paragraph), the authors say they use DBSCAN to measure scission efficiency. I think more details are needed here, such as how this is done (e.g. epsilon, Nmin values, etc.)

We have now added the information (eps=1.4, min_samples=1) on the parameters used for DBSCAN.

- There are other theoretical studies on how ESCRTs remodel membranes (e.g. Lenz et al. PRL 2009; Fabrikant et al. PLoS Comput Bio. 2009). If they consider it appropriate, the authors should consider discussing similarities/differences with these previous reports.

We thank the referee for pointing this out. Lenz et al. proposed the buckling instability model based on observation of overexpressed ESCRT spirals promoting membrane invagination, in which membrane deformation and tubulation is induced by the relaxation of the bending energy of a closely-packed, elastically-frustrated flat spiral filament. This was one of the first models on the topic, and an inspiration for our previous work, which we now discuss. The model did not consider the change in filament-membrane interactions upon buckling, or the active changes in the filament geometry which were recognized as important in the process only relatively recently. The dome model proposed by Fabrikant et al is based on observation of dome-shaped membrane deformation, and they have found that ESCRT-III mediated scission is spontaneous fast fission by estimating the filament-membrane binding affinity associated with the free energy differences in the fore-fission and post-fission states with the dome-sphere model. However, the presence of an ESCRT assembly in the membrane neck and its disassembly were not taken into account back then, which is now known to play an important role in fission.

Our simulations find, as also suggested in these two theoretical studies, that a large amount of energy is used to lower the fission barrier via membrane neck constriction, and once the neck is close to the fission limit, membrane scission appears to be spontaneous. However, there are substantial differences between the mechanism considered in our model, which includes active changes in the filament composition and geometry, and the previous theoretical reports. This is partially because a large amount of new biochemical and cellular data, and higher resolution images have been reported in the past decade to facilitate our modelling. We cite these two theoretical studies first in the introduction and then in section “Membrane scission is sensitive to constriction kinetics” when we discuss spontaneous scission to remind the audience of the previous theoretical work.

Reviewer #2:

This manuscript by Jiang et al. presents a computational investigation into the function of protein complex ESCRT-III, a ubiquitous machinery used by the cell to sever its membrane from the inside. This action is known to involve many distinct proteins that successively form several membrane-associated filaments with different shapes, resulting in eventual membrane rupture. Experimental information on this process essentially consists in static, high-resolution structural data and dynamic studies providing little spatial information due to their limited optical resolution. Combined with its complexity, these factors result in substantial uncertainty as to the mode of action of the ESCRT-III complex. This implies that a detailed understanding of the physical constraints governing this process has a potential to significantly narrow down the space of possible mechanisms and thus further our understanding of this crucial biological problem.

In their study, the authors design an elegantly simplified model of the ESCRT-III assembly and deformation sequence. They are therefore able to validate a physically consistent, detailed scenario for the resulting membrane severing action for the first time while providing many valuable insights into the mechanical requirements on the protein filaments involved. Given the current state of knowledge on this problem, I believe that this study strikes an excellent balance between the inclusion of biologically relevant details such as the succession of several filament species, and the necessity to simplify the problem to extract valuable insights as well as keep the number of unknown parameters under control. They also do a great job of keeping the discussion intelligible despite the relative abundance of such parameters. I wholeheartedly recommend the publication of this manuscript, and request that the authors will implement the following recommendations which I believe will help amplify the impact and usefulness of their work.

Detailed comments

- In the initial description of the polymer assembly sequence, specifying from the onset that R_2 is smaller than but close to R_1 would help the reader form an accurate early picture of the process.

Indeed, we have now done this.

- The approach chosen by the authors to investigate the influence of the various mechanical parameters is to choose a certain set of parameter values, and to then vary individual parameters

around these values while holding the other parameters fixed. Some parameters are not varied, such as the filament-to-membrane sticking energy. It would of course be unreasonable to expect the authors to explore the whole 7- or more-dimensional parameter space of their system. That said it would be nice if they could explain how they chose the central set of parameter values that they perturb around. Were these inspired by some measurements from the literature, or are they reasonable orders of magnitude inspired by similar systems? A discussion would be welcome, perhaps accompanied by a table of the model parameters and reasons for the choice of their values. In addition to the parameters that are already explicitly discussed, I would be interested in knowing how the initial filament length is chosen and whether the authors expect that much will depend on this parameter.

We thank the reviewer for this good point. The parameters in our model are the following: the radii of the three filaments, rigidities of the three filaments, the filament length, the strength of filament-membrane adhesion, and the diameter of the cargo particle.

While we are not modelling a specific system, the geometrical parameters that we choose are in the regime of physiologically relevant parameters. The target radii of the filaments (30.8 nm for Flat Spiral, 28.0 nm for Wide Helix) are based on the AFM measurements reporting Snf7 spirals to be ~20-50nm wide on membrane substrates (Henne et al 2012, Shen et al 2014), and cargo diameter (36.8 nm) is based on the reported ILV and exosomes diameters of 30-160 nm. The target radius of Tight Helix is chosen by a parameter scan, and it matches well the theoretical fission limit (~ 3nm, Kozlovsky et al 2002). The rigidities of the first two filaments are selected such that they yield the observed intermediate conical deformation in Pfitzner et al 2020, and for simplicity, we use the same stiffness for the third filament. Little is known about the strength of the membrane adhesion of these composite filaments. The filament-membrane adhesion is chosen as the smallest value that allows the filaments to remain stably attached to the membrane. This parameter has been tested previously in Harker-Kirschneck et al 2019 where the single filament model is originally developed. We have added a paragraph to discuss the choice of the parameter values in section S2 “Simulation protocols” (Supporting Information), subsection S2.1 “General settings”.

In terms of filament length, filaments shorter than one loop are not able to deform the membrane. Conversely, filaments that are very long form secondary spirals (see the snapshot below). The formation of secondary spirals, also seen experimentally *in vitro*, indicates that the preferred geometries of the filament subunits are more likely to be rings rather than spirals. For Tight Helix scission, we find the dependency on the polymer length to be more interesting. As

suggested in literature (Bashkirov et al 2008), very long filaments decrease the scission efficiency. We have thus repeated the same analysis of Fig. 3D with a shorter filament (i.e., decreasing the length of 103 monomers to 13 monomers, see Fig. S9 B), and we find the scission efficiency increases but the trend remains the same. We have also added a set of simulations using fixed final target radius for Tight Helix and varying the length of filament, and it shows clearly that scission efficiency increases for shorter filament lengths (Fig. S9 A), as they provide less stabilization to the neck.

We have added a paragraph to discuss the impact of the length of Tight Helix on scission efficiency at the end of section "Membrane scission is sensitive to constriction kinetics".

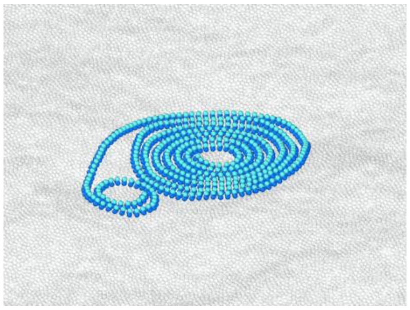

Fig.1. Simulation of an overexpressed Spiral (393 monomers) on a deformable membrane.

- Another related point is that the authors mostly discuss the values of their parameters in simulations units. This clearly makes for a cleaner paper overall, but providing a (putative?) dictionary between these and physical units somewhere would make it quicker and easier to relate these results to other studies, be they theoretical or experimental. One especially valuable piece of this discussion would be a better understanding of the typical time scales at which the transition pictured in Fig. 4B takes place.

We thank reviewer for this valuable point. In the original article, we have used nm as the default length unit but used τ for time as timescales are more difficult to map from coarse-grained simulations, and we also lack time-resolved experimental data for benchmarking. We decided to keep it like this, but we now provide the unit conversion for time and energy in the SI (subsection S2.6, Table S2).

- Time: $\tau \sim 0.02\mu s$. This is obtained by mapping the diffusion constant of the bead in the membrane model (D=0.06 σ^2/τ) to a lipid molecule in a bilayer membrane (D=20 $\mu m^2/s$, Fahey and Webb, doi: 10.1021/bi00608a016), which gives our MD time unit

$\tau \sim 0.018\mu s$. However, one bead in our model represents a patch of lipids, rather than a single lipid molecule, so this is a lower bound. A similar timescale is obtained when mapping the diffusion constant of our three-beaded protein unit in solution (D=0.25 σ^2/τ) to the diffusion constant of small soluble proteins (D=100 $\mu m^2/s$, Young et al, doi: 10.1002/bit.260220504), which gives $\tau \sim 0.013\mu s$. But again, our three-beaded unit does not necessarily represent a single ESCRT protein. Taking this data, the lower bound time scale associated with the transition in Fig. 4B is $\sim 50\mu s$.

- Energy. The energy unit at room temperature equals to $k_B T_{T=300\,K} \sim 4.14 \times 10^{-21} J \sim 0.6 kcal/mol$. We think it is more intuitive to use $k_B T$ when discussing energies.

- Fig. 2 & corresponding text: The authors state that an excessive value of k_2/k_1 or of R_1-R_2 leads to an unstable filament attachment. Couldn't this be compensated for by making the filament more sticky to the membrane? This is of course connected to the previous item - if a rationale were given for the value of the stickiness used in the simulations, then this wouldn't be much of a concern.

This is an interesting question. As discussed above, we do not have a reference value for the binding strength, but the binding strength should allow the filament to stick to the membrane, while also allowing it to relax on the membrane easily. In that sense, there is an upper limit on the binding energy that we can use. Above this, the adhesion causes the membrane to crystalize and the system's dynamics is arrested. We have found that limit to be $6k_B T$ in our simulations. Therefore, increasing the filament-membrane adhesion strength can increase the allowed range of mismatch in the target radii (I.e., the threshold $R_1 - R_2$ value will increase), but it will not change the qualitative mechanism.

- In Fig. 3C, the color bar measures E_memb in units of k_BT. It would make more sense to me to describe the membrane energy as an energy per unit surface since the membrane "beads" don't have a straightforward real-life counterpart. This would mean giving this energy in units of k_BT/σ^2 or k_BT/nm^2.

The current measurement is based on averaging the pair potential energy between each pair of beads. We agree that it would be great to report the local energy per unit surface, which we have tried to do, but we found the measurements of the local surface area in these highly curved geometries to be non-trivial and highly method-dependent, and to generally have large error bars.

This made the plots messy and the potential energy profile less clear compared to the energy per bead measurements. Therefore, we decided to keep the plot showing the energy per bead; it is more precise and it shows clearly that the location of membrane breakage is where the local potential energy is highest.

However, we were able to compute the local energy per area at the place where the membrane breaks. Detailed estimations are the following:

- With cargo: The pair energy per-bead at the location is $1.1 k_B T$. Surface area for one section/membrane bead layer in the neck is $S_{bin} = 2\pi R_{neck} \cdot \sigma = 2 \cdot 3.14 \cdot 5 \cdot 2.3 = 72.22\ nm^2$, average number of beads per circumference in the neck is ~30. Diameter per bead $\sigma = 2.3nm$, neck radius $R_{neck} \approx 5nm$. Therefore, the energy per unit surface area associated with membrane breakage is $\sim 1.1 \times 30 \div 72.22 = 0.5\ k_B T/nm^2$.
- Without cargo: The pair energy per-bead at the location is $2.4 k_B T$. There are only 2 beads in this section/membrane bead layer at the tip of the invagination. We estimate the area as the sum of their cross sections $2 \times \pi \left(\frac{\sigma}{2}\right)^2 = 8.31\ nm^2$, and the associated energy per unit surface area is $\sim 2.4 \times 2 \div 8.31 = 0.6\ k_B T/nm^2$.

We report the energy per unit surface area associated with the pore formation in the main text, section “Membrane scission is sensitive to constriction kinetics”, when we discuss Fig. 3C.

- p. 4 and Fig. 2A,B : the authors state that $k_1 \simeq k_2$ large is required for deformation, but the data of Fig. 2A,B suggests that $k_1 \ll k_2$ would also work. Could the authors clarify why they rule out this regime ? Is this because of the increased risk of a Fig. 2C-type detachment?

Indeed, $k_1 \ll k_2$ would create a strong deformation in a form of a tube, but not a long-lived conical deformation as observed in experiments (Pfitzner et al 2020). We have now clarified this point.

- The three-polymer model is nice and simple, but why not even fewer? I would be interested in having the authors quickly discuss whether they tried a "Flat spiral -> Tight helix" scenario and why it did not work.

This is a very interesting question. We believe the possible role of the Wide Helix could be to change the filament orientation and control the direction of buckling.

In more details, the reasons that we have Wide Helix and Tight Helix as two different filaments are the following: i) It is clear from in vitro experiments that the intermediate buckle deformation appears to be a stable, long-lived state. The long-lived Wide Helix is needed to capture this, ii) The Wide Helix is a filament that changes the orientation of the copolymer binding site to the membrane. The Tight Helix is a filament that constricts the membrane dramatically and mediates scission. The two helices appear to have different roles, which we wanted to explore, iii) Experimentally, there are ESCRT monomers that modify the co-polymer architecture from flat spirals to 3D spirals, and there are ESCRT monomers that promote scission. The first membrane invagination and second tubulation do not occur in a single step.

Alternatively, one could imagine the system with an initial polymer forming a spiral with a tilt, mid-way between the Spiral and the Wide Helix, followed by a Tight Helix, reducing the numbers of polymers to two, as is seen in many archaeal genomes, if there is an additional step that aids the recruitment of the first polymer (ESCRT-II in this case).

In a separate recent work (https://doi.org/10.1101/2022.05.10.490642), we coupled chemical kinetic and theory of elasticity to probe the dynamic stepwise assembly of these composite filaments, and explore the role of different monomers. We found that the free energy barriers for the assembly of the highly curved final filament needed for scission are lowered the more monomer types there are in the sequence.

We now comment on this point in the text.

- The "tight helix disassembly triggers fission" effect is perhaps the most interesting physical puzzle brought about by the simulations. This sounds reminiscent of a proposal for the function of dynamin by Bashkirov and co-workers (DOI: 10.1016/j.cell.2008.11.028), although I was never able to make intuitive sense of the physical mechanism at play in their model. Could the authors discuss their findings in relation to this more complicated model? This also sounds a bit reminiscent of the breaking mechanism of Ref. [29], which is also a bit unclear to me (despite

my being an author of the paper!). Does the authors' result shed new light on these earlier ideas? And regardless of earlier papers, can the authors speculate on how disassembly causes breaking?

This is an interesting point. Bashkirov et al postulated that rapid disassembly of proteins could destabilize the bilayer structure underneath, which would then be prone to breakage. In our simulation, the highly curved membrane neck is also stabilized by the adsorption between the protein and the membrane (Fig. S7), and this stabilization is removed upon filament disassembly, which leads to local membrane poration, and neck breakage. However, since the membrane model that we use does not include the fine details of bilayer structure, the instabilities arising upon protein disassembly in our simulation may not be the same as proposed by Bashkirov et al.

The model by Morlot et al. for fission by dynamin (Cell 2012) proposed that the local increase of elastic energy at the edge of constricted dynamin rings is sufficient to increase the probability of fission at the edge of constricted dynamin rings. This has been observed both in the case of dynamin rings, as well as in the case of ESCRT-III filaments in cytokinesis. In agreement with this model, in our simulations, we clearly see that fission occurs at the edge of the ESCRT-III coat. We rationalized this via the energy plots showing that the edges of the filaments are the point of the highest local energy increase.

We agree that the analogy between dynamin and ESCRT-III scission mechanisms is underappreciated, and all our simulation work thus far suggests that the two systems appear to have remarkably similar mechanisms. A more in-depth comparison would be in our opinion more appropriate focus for a separate paper, which is why we decided not to discuss the dynamic-ESCRT comparison in more details here.

- p.6 : "Finally, the location of the disassembled ESCRT-III monomers depends entirely on the disassembly sequence used" : this feels like it depends on the value of the diffusion coefficient used in simulation units. Again, it would be helpful to be able to relate these quantities to known orders of magnitudes in physical units.

Directly converting from coarse-grained units to physical units is not straightforward, which is why we used the membrane bead diffusion to perform the mapping to physical time-units, as discussed above. This yields protein diffusion coefficients within the physiological regime of globular proteins. However, it is important to add that a three-bead monomer in our filament does not directly map to a single protein, but rather to a filament section. Moreover, diffusion

coefficient of ESCRT proteins in reality depend on their local interactions with the membrane and other proteins in the vicinity, as well as their conformation, which is severely altered by Vps4-induced unfolding. So a direct comparison of time-scales between simulations and experimental systems (for which these parameters are also unknown) is not necessarily meaningful.

We agree that the partitioning of the protein will clearly depend on the relationship between the time-scales of membrane-sealing, filament disassembly, and monomer diffusion. We have not explored how changing this relationship affects the partitioning, as these parameters are also experimentally unknown. We cannot claim that the partitioning measured in simulations quantitatively maps to experiments, but we have no reason to believe that the general trends with respect to the protocol of filament disassembly would not hold. We have now commented on this in the text.

- Unless I have missed it, the authors never mention the spontaneous pitches of their two helices. This should be done somewhere. Is it zero as Fig. 1B suggests?

Indeed, there are no spontaneous pitches imposed and the target geometries of the filaments are rings. We have now made it more clear that those target geometries are tilted rings without pitch, in the first paragraph of the subsection S2.1 “General settings”, section S2 “Simulation protocols” in the SI.

Typographical errors & clarifications

- abstract: "We set to" -> "We set out to" done

- Fig. 3 caption: "for fast constriction, filament partially detaches" -> "for fast constriction, /the/ filament partially detaches" done

- Fig. 3 caption: "for slow constriction, filament remains attached" -> "for slow constriction, /the/ filament remains attached" done

- p.4 section title: "Rigidities of the Spiral and Helix should be comparable and large" -> "Rigidities of the Spiral and /Wide/ Helix should be comparable and large" (to avoid a possible confusion with the tight helix) done

- p.4: "The stiffer the Helix is, the deeper the membrane deformation" -> "The stiffer the Helix, the deeper the membrane deformation" done

- p.6: "The protocols [...] induce membrane scission relatively good" -> "relatively well" or "relatively efficiently"? "good" sounds a bit strange. Changed to "relatively well"

- p.6: "the two protocols [...] cut larger membrane necks than the randomized protocol" : a word is missing between "neck" and "than". "larger" was included earlier in the sentence.

- p.6: "the Did2-Ist1 complex bind" -> either "complexes" or "binds" Changed to "binds"

- p.7: "up to the smallest size" -> "down to the smallest size" done

- p.8: The axis coordinates "x" and "y" should be in italics/mathematical notation.

The axial labels of Figure S5 has been updated, so all figures have axial label in correct format now.

- "Time step of [...] is used" -> "A time step of [...] is used" done

- "Lammps" -> "LAMMPS"? done

- p.8: "rest of the system": do the authors mean the cargo, or is there something else that I am not seeing? We meant the cargo and the filaments. We have now clarified this.

- p.8: some intra-filament bonds appear to be Lennard-Jones (filament-filament binding) and others harmonic (top right of the page). Which is which is a little unclear. I think it would be useful to have a slightly slower, clearer description of what the interactions are between their many different types of beads. Maybe include a matrix-like table?

Thanks, we have now added Table S1 to summarize all the bead-bead interactions. The table is mentioned in subsection S2.1 "General settings", section S2 "Simulation Protocols" (Supporting Information).

- p.8" "tilted ring": I do not understand this term. Is this another name for a helix? Or is this the

same kind of objects as in Ref. [16]? In either case the value of the tilt parameter - pitch or angle - should be given. The term "tilt" also appears in p.9 without being more understandable.

Apologies for the confusion. It is the target ring geometry with tilt defined as in Harker-Kirschneck et al 2019, and we now define the “flat ring”, “tilted ring” using the original notation τ (see the beginning of the “simulation protocols”), which we hope elevates the confusion.

- p.9: "R_{init} to nm R_{final}": it looks like "nm" should be removed. Done

- p.10: The authors of Ref. [1] appear to be missing. Fixed

-- Martin Lenz

Reviewer #1: **No:** As far as I've seen, the codes for the computational model are not published or linked to in this manuscript.

The essential scripts for the simulations and analyses are available on github and University College London Research Data Repository. We have now added the link in the main text (section “Data availability statement”).
